# A Qualitative Study of Drug Treatment Conformity Behavior among Young Drug Users Who Are in Recovery in China

**DOI:** 10.3390/ijerph192214832

**Published:** 2022-11-11

**Authors:** Chen Li, Guandong Song

**Affiliations:** School of Humanities and Law, Northeastern University, Shenyang 110167, China

**Keywords:** youth, drug addicts, drug treatment conformity, qualitative research

## Abstract

In response to social concerns about young drug users, this study aimed to qualitatively explore the types of drug treatment conformity behaviors and the processes of behavior formation among this population. Twenty-one young drug users were selected through purposive sampling, in-depth interviews were conducted using a semi-structured approach, and the data were then analyzed. The social conformity theory is used as the framework of the analysis, and the results indicate that drug treatment behaviors can be differentiated by the three types of motivation that produce conformity with drug treatment: the cognitive, affective, and utilitarian. These three types of motivations produce three types of conformity, respectively: drug treatment abidance, drug treatment compliance, and drug treatment obedience. They are affected by informational social influences, normative influences of significant others, and normative structural levels, respectively. We also propose a model of the information processing involved in drug treatment conformity. Based on an in-depth analysis of the characteristics and formation processes of the three different types of conformity, intervention strategies are proposed. This study has important guiding significance for helping young drug users maintain their drug rehabilitation ethics and successfully return to society.

## 1. Introduction

Drug addiction is a violation of the law with serious consequences for the individual, family, and society [1], and drug addiction has a complex physiological, psychological, and genetic basis, being a product of the interaction of various factors among humans, drugs, and society [2]. Moving from addiction to physical, psychological, and social rehabilitation requires multiple systematic and complex interventions, and many disciplines such as medicine, sociology, psychology, as well as administration are looking for ways to help drug users address relapse [3]. However, although the physiological cessation of drug effects can essentially reach 100% after detoxification, the relapse rate remains high, and the drug problem recurs [4]. A person’s exposure to drugs usually begins in adolescence, and thus the youth population is a group vulnerable to drugs [5]. Youth is a critical stage in the socialization process, and at this time a drug user can become a social outlier, subject to rejection and discrimination in all aspects of life, employment, and marriage. This rejection can lead to a tendency for young drug users to refuse treatment interventions and, after failing to return to mainstream society, revert to associating with drug-using sub-cultural groups. Therefore, the research to date has focused on how to help young drug users maintain their drug treatment motivation and behaviors. A prerequisite for addressing this issue is to understand the mechanisms and factors that influence drug users’ drug treatment conformity behaviors.

The Chinese government has been emphasizing drug prevention and rehabilitation efforts for youth [6]. In China, there are three models of drug treatment: voluntary, compulsory isolation, and community-based. Voluntary drug treatment is conducted by medical institutions, while the other two, compulsory isolation and community drug treatment, are government-led, in which public security authorities, the judiciary, and local governments play different roles and introduce various intervention policies and strategies [7]. Community-based drug rehabilitation is a new form of anti-drug treatment strategy since the introduction of the Chinese anti-drug law in 2008, enabling drug users to gradually achieve rehabilitation in their own family and community environment without being separated from society. During the community drug rehabilitation process, drug users are supervised by anti-drug social workers and undergo regular urine tests to make sure they have not relapsed; if they are found to have relapsed, they will be determined to be severely addicted to drugs and will face 1–3 years of mandatory isolation.

Scholars have carried out research on the causes and processes of behavior during drug treatment using different theoretical models. From the perspective of social learning theory, Marlatt and Gordon have argued that relapse after drug treatment is an overlearned pattern of bad behavior which is caused by negative behavioral habits, and that individuals are prone to relapse when they encounter dangerous situations because they have not learned how to use alternative adaptive behaviors [8]. Prochaska and Norcross proposed a five-stage model of change, in which the five stages are the pre-contemplative period (denial of the problem and resistance to change); the contemplation period (thinking about change); the decision to act period (forming a decision to change); the action period (changing the original behavior and environment); and the maintenance period (maintaining the new behavior) [9]. Gloria Chan et al. explained, from the perspective of self-determination theory, why drug users decide to quit [10]. They suggested that the interactive mechanism that determines whether or not a person uses drugs is the psychological need for relationships, with meaningful interpersonal relationships and intimacy being the most important determinants. The same is true for the process of drug treatment, in which the satisfaction of psychological needs becomes an important intrinsic motivation for drug addicts to quit. Studies have shown that the presence and intensity of motivation is one of the key factors affecting the success of drug addicts in detoxification [11,12]. Zhang et al. investigated the motivation of heroin addicts to enter a drug treatment facility and summarized six types of motivation for detoxification. With the exception of the sincere detoxification type, all of these types of detoxification can be described as “impure” and will have a negative effect on maintaining integrity [13] Gao interviewed drug addicts and concluded that synthetic drug users often decide to quit based on multiple factors rather than a single factor, including a series of serious consequences of drug use, the occurrence of major events, pressure from family and household members, and fear of forced withdrawal [14].

Unlike the theoretical perspectives used in previous studies, this study adopts social conformity theory to examine the types of motivation and behavioral processes involved in drug treatment. Conformity refers to externally consistent behaviors that are influenced by social information; people usually want to align their attitudes, opinions, and behaviors with the majority, a phenomenon known as “social conformity” [15,16]. When people behave differently from the majority, they become suspicious of themselves, feel anxious or embarrassed, and adjust their behavior to conform to social norms or group opinions [17]. Since Asch’s wandering vision experiment [18], many classic social psychology experiments have demonstrated that individuals will adjust their initial behavior to conform to group opinion when there are differences between them and the group [19,20,21]. Deutsch and Gerard proposed the classical dual-motive scheme based on the improvement of Asch’s experiment, namely normative and informative social influences [22]. Different scholars have different opinions on the types of conformity and the reasons for the generation of conformity. Cialdini et al. considered different motives for the generation of social conformity behavior which they divided into three main categories: the collection of external information to ensure the consistency of one’s own behavior; conformity to social norms or others’ expectations to ensure the rationality of one’s behavior; and compliance with commitments to maintain a favorable self-concept [16]. Saul et al. argued that due to the different social effects of pressure, these motives can be specifically divided into conformity, obedience, and compliance. Conformity is an unconscious, automatic process of choosing to act in agreement with others in response to pressure from others; compliance is a change in behavior in response to a request from others; and obedience is a change in behavior in response to an authoritative command from others [23]. Nail proposed a convergent perspective in which conformity is divided into three types: compliance, obedience, and acceptance [24]. Compliance is insincere and is only an external form of behavior to comply with another person’s request. Obedience is submitting to an explicit command to obtain a reward or to avoid punishment. Acceptance is a sincere decision to comply with another person. Song, Guandong, et al. further revised the definition of conformity to mean that it is a consistent behavior resulting from the influence of social information. At the same time, conformity has been studied by dividing it into external and internal forms, and it is believed that both external and internal conformity are manifested as the three types of abidance, compliance, and obedience, with subordination being a special form of conformity [25].

Young drug users are clearly a heterogeneous group that strays from social norms, and their deviation from social norms leads to rejection, which is a form of social punishment [26]. When they feel stimulated by social messages in their daily social interactions, they also develop the idea of being consistent with the normal population, because consistent attitudes and behaviors enhance the possibility of being accepted by society [27]. Everyone has acceptance needs, and the idea of being accepted becomes the driving force behind the emergence of drug treatment behaviors, which leads to changes in drug use behaviors and the emergence of drug treatment conformity behaviors. Some studies have shown that supportive and motivating community relationships, meaningful activities with peers, and distance from recovery-impeding communities were identified as important recovery components [28]. Youth that have a greater neighborhood sense of community are believed to be less influenced by negative environmental experiences and less inclined to engage in drug and alcohol use [29]. In addition, drug use behavior is a behavior of conformity to the drug use subculture [30], and once an individual enters the drug use subculture, he or she is influenced by various messages and will develop behaviors that are consistent with the group, creating drug use conformity. Social conformity theory can likewise be used to enhance drug users’ motivation to quit. Some studies have shown that motivation is a key factor in determining whether or not a drug addict will relapse, and that extrinsic drug use behavior is driven by intrinsic motivation that can lead to a change in behavior [31]. There is a significant negative correlation between the motivation to quit drugs and the propensity to relapse, and individuals are motivated to quit drugs by the consideration of whether or not quitting will satisfy their physiological, psychological, and social needs caused by intrinsic or extrinsic stimuli [32]. Changes in the behavior of young drug users can be achieved through different forms of motivation for drug treatment conformity.

In research to date, most of the studies on the drug treatment process have focused on the factors influencing the drug use and relapse process and have expected to achieve prevention by controlling these influencing factors. Thus far, most of the research has been conducted at the level of the psychological motivation of drug users, using a top-down quantitative research approach, and less research has been conducted at the level of social psychology, using a bottom-up research approach to explore their behavioral patterns. This study aims to develop a model of the types and influences of the motivation and behavior of young drug users and to answer the following research questions: (1) What are the processes that produce drug conformity behavior among young drug users? (2) What are the types of conformity behaviors among young drug users? (3) What are the influential factors that lead to the development of conformity behaviors among young drug users? It complements the research gap on the drug treatment process for young drug users if they can explain why it is currently difficult to maintain drug treatment ethics and why high relapse rates occur, and explores interventions to improve the drug treatment and rehabilitation process of young drug users.

## 2. Materials and Methods

### 2.1. Participants and Procedure

In this study, 21 young drug users under the age of 35 were selected for in-depth interviews as a way to explore the factors influencing their drug treatment conformity. The participants were drug users who had undergone community-based drug treatment in mainland China according to the Drug Treatment Regulations, which mandates the treatment and supervision of drug users in their original living communities. A “purposive sampling” approach was adopted to ensure the representativeness of the sample, and a high degree of heterogeneity was selected within the specified age range (18–35 years). This ensured that the sample was typical of the subject population and thus provided the greatest range of initial information. The sample was comprised of persons who were mentally and intellectually normal, had a history of drug use, had a history of drug rehabilitation, and had returned to society (See Table 1). Data collection took place in the first half of 2022. The Ethics Committee of the Northeastern University of China approved this study.

This study used semi-structured interviews to obtain its research data. A rough interview outline was used to clarify the direction of the interviews and to help understand the drug rehabilitation process of the interviewees. Face-to-face interviews were conducted and interviewees were informed of study confidentiality. Data collection consent forms were signed after interviewees were consulted. The interviewers took notes on key points and recorded the interviews with a voice recorder. During the interview process, the content was adjusted or additional questions were added in a timely manner according to the interviewees’ individual narratives, and self-reflection and follow-up questions were continuously conducted. The interviews were conducted at the community drug rehabilitation center, and each interview took an average of 30 min. During the interviews, the following guiding questions were asked: “Tell us about your drug rehabilitation process?” “What was your motivation for detoxification?” “What do you think happened during the detoxification process that had a big impact on you?” “What support did family or friends provide to you?” “How has your life changed while using drugs or after recovery?” “How has your self-evaluation changed since detoxification?”, etc. After the interview, the information obtained was transformed into textual material and coded for analysis.

### 2.2. Data Analysis Method

A theory-driven thematic method based on social conformity theory was used to analyze the data. The analysis used Nvivo12 software(QSR International Pty Ltd., Melbourne, Australia) to transform and code the interview material. To ensure the reliability of the study, three researchers were selected to conduct independent coding in the initial open coding stage, and a comprehensive analysis was conducted after completion. The analysis of the data was conducted according to the following procedure: (1) The audio files were organized and converted into textual information. (2) Three coders were selected and trained. The three coders were an anti-drug psychology graduate student, an anti-drug social worker, and an anti-drug college teacher. (3) Holding a training session for coders. An initial coding protocol was established using three interview transcripts as cases. (4) The files were open coded to make it possible to define the meaning of the source material by organizing and summarizing it and coding the content of the material word by word to be able to conceptualize the content. (5) At the end of the initial coding, the three coders were organized to conclude through discussion and analysis that the categories were conceptualized and summarized by identifying the concepts and also refining the material. (6) Saturation tests were conducted by coding the last three remaining interview reports to identify categories that were not mentioned. (7) The three coders came together and finally defined and named the categories by analyzing and reviewing if there were any topics not covered. In this study, 54 initial concepts were coded, and 11 categories were obtained after merging and summarizing: ultimately, “cognition and experience,” “emotion and support,” and “reward and punishment” were summarized. According to the theoretical guidance, they correspond to the following: “drug treatment abidance”, “drug treatment compliance”, and “drug treatment obedience”.

## 3. Results

### 3.1. Introduction of the Themes

Based on the framework of social conformity theory, three theoretical concepts of cognitive, affective, and utilitarian dimensions were selected as themes to guide the researchers’ search for meaningful data on motivations for drug treatment conformity. These concepts were extracted based on the following codes to explain how substance abusers are able to maintain drug treatment conformity behavior. Coding structure of narratives of participants are shown in Table 2.

### 3.2. The Process of Generating Drug Conformity Behavior among Young Drug Users

When asked about their experiences with drug addiction, participants’ responses all centered on a specific storyline from the beginning of drug use to attitude change and then to behavior change. This storyline was comprised of three parts: (1) Changes in social information occurred after initial drug use. Respondents’ perceptions of drugs generally changed after they began using drugs (N = 21). Prior to drug use, individuals were often curious because they did not know about drugs (N = 17), or because of peer pressure within subcultural circles (N = 12) (e.g., 03: “If I don’t do drugs, they will definitely not call me at future parties”), or because they were influenced by social emotions (e.g., 09: “I had just broken up at that time and was so upset that I wanted to get myself drunk at the bar.”) Or, they may have reported purposeful drug use (e.g., 18: “I broke my cervical vertebrae in a car accident, and this helps with the pain, so it doesn’t hurt anymore.”) However, after being exposed to drugs, the veil of mystery was lifted and their perception of drugs was directly influenced by their own physiology and psychology, overturning their original perceptions. Respondents perceived adverse physiological reactions to drug use, negative psychological effects, and psychological burdens when engaging in social interactions as changes in social information resulting from the act of taking drugs.

**Figure 1:**
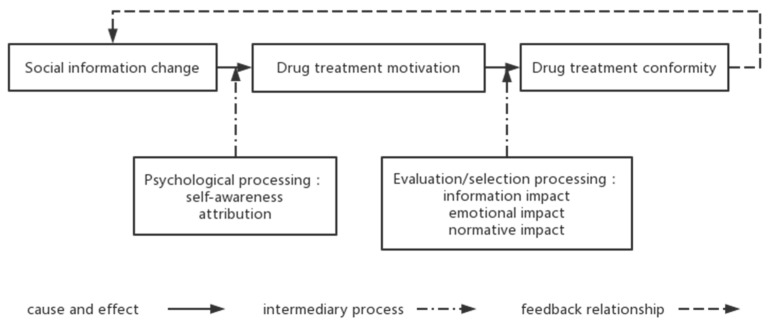
The development of drug treatment conformity among young drug users.

(2) The mental processing of social information is influenced by factors related to the drug users themselves. Social information processing theory suggests that an individual’s behavior changes because of changes in the context in which he or she lives, and that the social context in which people live is constructed by the cognitive processing of information [33]. Different individuals will interpret the changes in social information resulting from drug use differently due to differences in their life environment and experiences, and at this point the triggers of the external context will be transformed into the processes needed for self-cognition, such as receiving education about the dangers of drugs (e.g., 12: “I really didn’t know that drugs were so harmful, I thought it was just for fun, I didn’t know it would become addictive, and now that I know the harm, I don’t want to touch it anymore”), or the young drug user has been advised by his or her family (e.g., 19: “Every time I go back to my parents’ house, I have to be advised, and my wife tells me that if I don’t quit, I will lose my family”), or the young drug user has been punished by the law (e.g., 21: “I have been in rehab for two years, and I couldn’t find a good job after I got out. I really learned my lesson.”) The individual is now in a state of arousal, they recognize the impact of drugs on their lives from a conscious, emotional, and utilitarian perspective, and this state of arousal directs behavior toward the chosen goal.

(3) The drug treatment behaviors of young drug users are conformity behaviors influenced by social norms and information. They are internal attribution processes that are constantly deepening and changing. Individuals evaluate their behavior based on their belief systems, self-efficacy, and information received from those around them, and respond by choosing behaviors that will maintain their good self-concept [34]. The need to conform to social norms is driven by the altered cognition and utilitarian thoughts of young drug users who tend to avoid harm, as well as the emotional support they receive, thus driving the emergence of drug treatment behaviors. When such users begin to detoxify, these triggers continue to act on them during the detoxification process, reinforced by feedback, and drug treatment conformity behaviors are thereby formed. When interviewees were asked about the process of detoxification, they first mentioned stimulus events in the external environment (e.g., 03: “I didn’t know drugs were illegal and harmful until I got caught”), then their perceptions of the events, and then what internal motivations arose as a result of the stimulus events, leading ultimately to the detoxification behavior and causing it to be continuously maintained. The development of drug treatment conformity among young drug users are shown in Figure 1.

### 3.3. Types of Motivation and Factors Influencing Drug Treatment Conformity Behavior among Young Drug Users

The analysis performed in this study revealed that the social conformity behavior of young drug users is purposive behavior governed by motivation. The motives that lead individuals to a particular behavior may be diverse, but there are necessarily dominant motives, and analysis of the interview results revealed that the motives for conformity behavior can be divided into the following three types: drug treatment abidance, drug treatment compliance, and drug treatment obedience.

#### 3.3.1. Social Information Enriches Perceptions, Leading to Drug Treatment Abidance

An individual’s behavior changes because of changes in his or her environment: the environment provides a variety of information, and the individual’s interpretation of that information determines what attitudes and behaviors he or she will subsequently choose [35]. From the interviews, it was found that inaccurate perceptions of drugs were the main cause of addiction among drug users (N = 17). “I didn’t know what it was before, my friend gave it to me and I used it, now I got caught and the officer told me that it is illegal to take drugs and I realized that it is bad,” (Participant 06). “The people I used to play with told me that this stuff is not the same as the heroin in the past, and that it is not addictive and will be fine if I use it,” (Participant 08).

Individuals usually have access to only limited information when making decisions, and individuals can make the best decisions after they have integrated social information into their consciousness [36]. Therefore, after having taken drugs, the stimulation of normal social interactions and the integration of social information promote a more comprehensive perception of drugs and are important reasons behind the motivation to quit drugs. Once they have been seized by public security authorities and gone through mandatory isolation and community rehabilitation, drug users have received basic knowledge about drugs and their hazards and general legal knowledge in the prison or community drug rehabilitation center, which changes their perception of drugs. “The police told me that this stuff is very harmful to people’s bodies and that they will be forced to isolate and be locked up when they are caught,” (Participant 18). “Every time I come to urine test, the social worker will tell me about the danger of drugs, and sometimes I also participate in their publicity activities, and I know that this stuff is too harmful, and I am determined not to touch it anymore,” (Participant 21).

Some people know that drug use is harmful to their bodies, but they think such claims are just exaggerations because they and their friends around them use drugs yet appear to be fine. Seeing cases of prolonged drug use in prisons or community drug rehabilitation centers and real exposure to cases of physical and mental illnesses due to drug use has an impact on their perceptions. “There are often educational classes in rehab that talk about the dangers of drugs. I’ve seen some people who have been in rehab for more than a decade, and the damage that drugs can do to people is irreversible, and some of them are crazy, not like normal people. I don’t want to become like that, I must quit,” (Participant 02).

Some drug users feel changes in their bodies and have bad experiences, especially after severe addiction, and physical illnesses caused by drug use begin to appear, making the experience of drug use worse. This, too, leads to a more accurate perception of drugs and thus more motivation to quit. “After I took drugs, my body was not good, my brain was not enough, and I was mentally exhausted,” (Participant 05). “After I used a few times, I couldn’t sleep afterward, I was nauseous, I was sleepy when I didn’t use drugs, and I was very tired,” (Participant 08). “I had no health problems at all, but after I finished using drugs, I was lazy every day and didn’t do anything,” (Participant 13).

Many theoretical studies have shown that awareness of self-induced behaviors is more likely to elicit change than awareness of behaviors induced by others [37], meaning that changes in drug cognition and experience trigger an intrinsic desire to change the status quo and lead to the development of more positive drug treatment behaviors. Thus, more emphasis should be placed on drug prevention education aimed at enhancing intrinsic motivation. Such drug treatment conformity behaviors resulting from the correction of drug cognition and changes in drug experience can be summarized as drug treatment abidance.

#### 3.3.2. Relationship Needs Influence Emotions, Leading to Drug Treatment Compliance

Acceptance into a social group is driven by individuals’ behavior, and following group norms is an effective way to gain that acceptance [38]. The need for social acceptance and maintenance of social relationships is also an important factor contributing to drug treatment conformity behavior. During the interviews, most participants mentioned the influence of their families on their motivation to quit drugs. Some mentioned family members’ admonitions and supervision of their drug use behaviors, which restrained them through social support forces. “When my parents found out, they kept advising me that I couldn’t use drugs, and they were particularly strict with me, asking where I was going when I left the house, and sometimes checking on me,” (Participant 13). Some people are motivated to quit drugs because their family members are very worried about them, causing them to develop a sense of guilt and remorse. “In rehab, when I ran the event my family came to see me. I could see from their eyes that they still had expectations of me, and although my family would not blame me, I felt that I really couldn’t touch this thing anymore. I was sorry for them,” (Participant 01).

Others experience major events and changes in family relationships, such as the breakdown of marital relationships, parental illness, and worries about the future of their children’s schooling and employment. Such major events upset the original balance of the family and make drug users aware of their social roles and the accompanying responsibilities they bear. For example, participant 01 had a history of 8 years of drug use, was a heavy addict, and had gone through 2 years of mandatory isolation. He stated during the interview that the most important point in maintaining his motivation to quit drug was his fear of the impact on his children’s future. “I was easily anxious before. After I use drugs, my temper gets worse, always angry, and then my wife found out that I used drugs and divorced me. My parents also advised me, told me that my child went to school, if he wants to be a soldier or something, I have a record here that will certainly affect him.” Participant 20 mentioned the impact of his mother’s illness in the interview. “This matter let the family know, originally that a while after my mother was just found out to have lung cancer, I was particularly depressed, the psychological pressure is particularly large, my mother has been monitoring me, I was determined to let her rest assured, I can no longer let her worry about me.”

All of the above statements came from drug users trying to meet the expectations of their families and to be emotionally altruistic rather than primarily self-interested. Altruistic behavior is behavior that has the goal of increasing the welfare of other individuals [39]. From the perspective of drug users, the outward manifestation of their altruistic motivation reflects a predominance of negative emotions such as guilt, shame, sadness, grief, anxiety, fear, and pain. These negative emotions lead to a strong motivation to abstain from drug use in order to circumvent them [40]. During the interviews, many drug users expressed the idea of quitting drugs completely because of the guilt they felt towards their parents, spouses, and children. They wished to avoid making these family members worry about them or to prevent their problems from affecting the lives of their families.

In addition, if there is more social support and more meaningful interpersonal relationships in the family or their living environment, the more likely it is that the addict’s drug treatment behavior will be compliant. Compliance refers to behaviors and attitudes that individuals generate through generalizations, judgments, and reasoning about the behaviors or attitudes of others in order to be consistent with their expectations, without necessarily agreeing with them internally [41]. Individuals are psychologically programmed toward the repayment of gratitude, and the more social support they receive, the more likely they are to care about the feelings of others and display a higher frequency of altruistic behavior [42]. The more emotional support and resources drug users receive from their families, peer groups, communities, and social organizations, the more they will perceive that having good and meaningful interpersonal relationships will create a sense of return to the group, thus promoting their compliance behaviors. This compliance behavior resulting from altruistic reasons such as emotional and environmental pressures can be categorized as drug treatment compliance behavior.

#### 3.3.3. Rule Control Affects Utilitarianism, Leading to Drug Treatment Obedience

A distinctive feature of the way in which young drug addicts describe the psychological changes that they experience during drug rehabilitation is that they are generally motivated to quit after being caught and punished for their drug use. According to the Narcotics Control Law of the People’s Republic of China, the first or second time they are caught, they are subject to administrative detention and community drug rehabilitation, while if they are caught for a third time, or if they show a serious drug addiction or seriously violate the community drug rehabilitation agreement, they are subject to compulsory isolation for a period of 1–3 years. The interviewees were all young drug users who had received community drug treatment, community rehabilitation, or mandatory isolation, and all answered the question, “Why did you start the program?” In response to this question, all interviewees said that they were caught by the public security authorities (N = 21) and mentioned that they would not come into contact with drugs again because they were afraid of being subjected to mandatory isolation (N = 21). The main reasons they gave for their fear were that they did not want to lose their freedom, that they feared being discriminated against by society, and that they feared the negative effects their drug use could have on their families.

“I only received community drug treatment. Although there was no restriction on freedom, every month I came to the Public Security Bureau to report for urine tests. I have a psychological burden in that when it is time for urine test, I’m afraid,” (Participant 05). “I entered the drug rehab at 2019 and started to detoxify and stayed for two years. I knew that drug use was bad, but I had been in this environment, and after I entered rehab I got out of it and slowly quit. If I had to rely on myself, I wouldn’t have the perseverance or control. After I got out, I vowed that I would never use drugs again, that I would never be forced to quit again,” (Participant 01). “If my child knows that I took this, how will he look at me? It also has an impact on the future of the child. It’s my fault for using drugs, and the result is retribution on my children,” (Participant 12).

**Figure 2:**
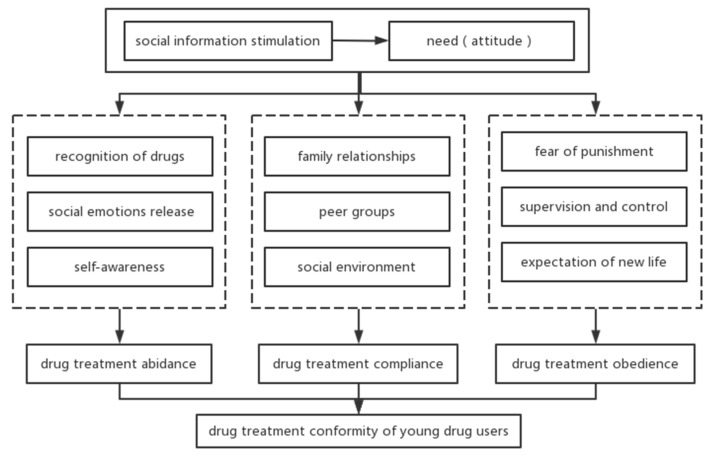
Factors influencing drug conformity behavior among young drug users.

Social control theory suggests that individuals follow rules because they are controlled by social norms. Social control is a mechanism that regulates the behavior of individuals and groups, leading them to comply with the rules of a particular social group [43]. Social learning theory similarly posits that people’s behavior arises because they wish to receive a reward or avoid a punishment, and that wanting to change a behavior or make it subside requires increasing the punishment or losing the reward, thus changing the individual’s expectations and altering their behavior [44]. Similarly, the avoidance of drug use and the enhancement of drug treatment conformity are the results of the punishment of drug use, which makes the individual aware that he or she is subject to social rules and that he or she must submit to them in order to avoid punishment or to obtain the expected normal life. The human instinct is to “avoid harm and get benefit”. This kind of conformity behavior, due to the utilitarian motivation of drug rehabilitation, can be summarized as drug treatment obedience behavior. Factors influencing drug conformity behavior among young drug users are shown in Figure 2.

## 4. Discussion

### 4.1. Discussion and Implications for Practice

The drug treatment conformity behaviors of young drug users can be classified into three types, depending on the motivation that produces the behavior: abidance, compliance, and obedience. All of these conformity behaviors are rational behaviors that are consistent with the individual’s attitude or behavior [45]. The objects faced by young drug users range from the micro to the macro level and include a whole social-ecological system of individuals, organizations, society, and the environment: the individual’s past experiences, his or her family members, peer groups, schools, companies, communities, social policies, laws and regulations, and cultural environments. Although their behavioral performance is forced by the role of various factors in the object, they all show consistency, but their subjective will is not necessarily the same as their behavior. For example, if a drug addict engages in drug addiction abidance, he or she can recognize the harm of drugs, and then will engage in the behavior of active drug refusal, and thus his or her subjective motivation will be stronger. If the drug user develops drug treatment compliance behavior because of emotional factors or relational pressure and the fear of negative effects on his or her loved ones, then his or her behavior is likely to be passive and may not be consistent with his or her perceptions. If a drug addict develops drug treatment obedience behavior because of fear of punishment and mandatory isolation, it is highly likely that that behavior will be subjectively passive. His or her emotional experience will be negative, and the behavior may not be consistent with the cognition. It can be seen that recognition of abidance is primarily motivated by internal influences, whereas compliance and obedience are primarily motivated by external influences.

These three models of social conformity are not static and do not exist in isolation. Each person may exhibit one or more of these conformity patterns at the same time, and several patterns may be transformed into each other or coexist at the same time. An example would be a drug addict who, after being punished, exhibits obedience. There may also be submissive behavior due to fear of adverse effects on the family. After being educated by the public security and judicial authorities, he or she realizes the danger of drugs to the individual, family, and society, and adopts a conscious abidance behavior.

The results of this study can serve as a guide when correctional authorities carry out educational correction. Correctional authorities can adopt targeted approaches to consolidate the motivation and conformity behavior of rehabilitated drug addicts when conducting education and management according to the different types of conformity behaviors. Since individual conformity behaviors are not static and multiple types of conformity behaviors may exist at the same time, attention should be paid to the dynamic nature of conformity behaviors.

There is a lack of knowledge about drugs and a need to strengthen young people’s scientific understanding of drugs in a comprehensive fashion. In drug prevention education, care should be taken to use a scientific and rational approach, rather than using “scare” education [46]. It is important to emphasize not only the breadth of the information that is disseminated, but also its comprehensiveness and scientific nature [47], to enable young people to translate that knowledge into the ability to refuse and resist drugs, and to internalize it as a code of conduct for healthy, positive living.

For emotional expectations, it is important to help drug users to improve their social support system. Social support is the material or spiritual help that individuals feel they receive from the state, organizations, or individuals to help improve their plight and restore their social functioning [48]. The social support system of drug users includes family, peers, the community, school, employer, and even policies and institutions. According to the results of this study, family support is the core influencing factor in the generation of drug treatment compliance [49]. We found that the stronger the social support, the more pronounced the drug addiction compliance behavior produced, especially among those who were married and had children. This is because the system, although an external stimulus, is most effective when it initiates the internal motivation of drug users. This, coupled with the fact that drug users are, due to loss aversion mechanisms, emotionally reluctant to lose their existing normal social relationships and social support [50], constrains their behavior, thus producing drug treatment compliance.

Finally, from a utilitarian perspective, the concept and mechanism of control should be implemented. The current drug governance framework in China is based on a governance model in which administrative coercive penalties under the legal framework are dominant and treatment and rehabilitation under the public health framework are complementary [51]. The concept of the policy is very humanistic, treating drug users as a trinity of “patient, victim, and offender,” but in terms of implementation, it is difficult to put the policy into practice. Only by effectively implementing the concept of control and institutional measures can we truly start rehabilitated drug users on the road back to society.

### 4.2. Limitation

This study has some limitations. We defined young people as those under 35 years of age, and indeed the scope of the study population is relatively broad, and possibly there are significant differences in the social environment and psychological aspects between emerging adults and those over 25 years of age.

The credibility of the results is arguably limited by the use of interviews to collect information, as participants may feel compelled to respond in a socially acceptable and desirable manner if they perceive that the interviewer (Author1) is a teacher of Criminal Investigation Police University of China. This is especially true if the study participants felt the need to impress the researcher in a way that was favorable to their image, despite the fact that the researcher made assurances to the participants that their information would be confidential. However, there is still a possibility that the above situation may occur. Since participants were selected through purposive sampling, there is a possibility that the transferability of the study results may be affected.

Moreover, both the legal and treatment paradigm in China may be vastly different from that of other nations, and it is possible that quite different results would emerge from a cohort of people in recovery in another country with different laws and treatment paradigms.

## 5. Conclusions

In this study, we focused on the experiences of 21 young drug users in quitting drug addiction and presented the developmental dynamics, types, and influencing factors of young drug users’ conformity behaviors. The formation of conformity to drug treatment among young drug users follows the path of “information stimulation-attitude change-behavior maintenance”. According to the different attributions of attitudinal change, conformity behaviors can be divided into three types: drug treatment abidance, drug treatment compliance, and drug treatment obedience. The main influencing factors of drug treatment abidance are the recognition of drugs, social emotional release, and self-awareness; the main influencing factors of drug treatment compliance are: family relationships, peer groups, and social environment; the main influencing factors of drug treatment obedience are: fear of punishment, supervision and control, and the expectation of a new life. These types of conformity behaviors of drug addicts can transform into each other or co-exist with each other. Based on an in-depth analysis of the characteristics and formation processes of different types of drug treatment conformity, intervention strategies for drug treatment conformity are proposed. Namely, these are to strengthen drug prevention education for adolescents in terms of addressing the deficiencies in drug knowledge; to improve the social support system for drug users in terms of addressing emotional expectations; and to implement control concepts and mechanisms in terms of addressing the utilitarian perspective. 

## Figures and Tables

**Table 1:** Demographic data of participants (N = 21).

Variable	Group	N	%
Gender	Male	16	76.2%
	Female	5	23.8%
Age	18–25	6	28.6%
	26–30	4	19%
	31–35	11	52.4%
Educational attainment	Middle school	4	19%
	High school	12	57.1%
	College	3	14.3%
	University	2	9.6%
Employment status	Unemployed	6	28.6%
	Employed	15	71.4%
Marital status	Single(Never married)	13	61.9%
	Married	2	9.6%
	Divorced	6	28.5%
Type of drug they used	Methamphetamine	15	71.4%
	Heroin	2	9.5%
	Cannabis	2	9.5%
	Tramadol	1	4.8%
	Polysubstance use	1	4.8%
Type of drug treatment	Previously undergoing voluntary drug treatment	1	4.8%
	Previously undergoing force isolation treatment	3	14.3%
	Only undergoing Community-based drug rehabilitation	17	80.9%
The number of years they have drug treatment	Less than 1 year	3	14.3%
	1–3 years	7	33.3%
	3–5 years	5	23.8%
	5–10 years	4	19.1%
	More than 10 years	2	9.5%

**Table 2:** Coding structure of narratives of participants.

Paradigm	Categories	Subcategories
Cognition and experience ↓ Drug treatment abidance	Awareness of the dangers of drugs	→Understand the harm of drugs/Drug use is illegal/Experienced the feeling of drug addiction and felt no fun
	Physical sensations	→Can’t sleep and irritability after taking drugs/After taking drugs, I feel inhumane/Highly stimulated/Slow brain response/I just want to do drugs and nothing else/Indulge in entertainment/Muddleheaded/Lack of energy without drugs
	Behavior change	→The habits of life became better after quitting drugs/Start thinking about their own future/Life is fulfilled/Not going to the entertainment places
Emotion and Support ↓ Drug treatment compliance	Family Influence	→Feel sorry for their own parents/Impact on their children’s future/Get divorced/Parental exhortation/Don’t want parents to worry about them/Can’t let their children be implicated/Strict parental discipline
	Stay away from bad friendships	→Drug-related friends all deleted/Reject the temptation of drug addicts
	The influence of normal friends	→Fear of friends knowing they are drug addicts/Hiding from friends/Admonition from close friends
	Lack of economic support	→No money for drugs./No money for normal life/Drug addiction causes a lack of desire to work/Incapacity for work
	Self-evaluation	→When using drugs all they want to do is take drugs every day/Feel like a loser when they’re on drugs/Being understood and helped makes them feel respected
Reward and punishment ↓ Drug treatment obedience	Scruples about punishment	→Life has a stain and fear of future impact/Regularly report to the Public Security Bureau with a psychological burden/Fear of two years of mandatory drug treatment isolation/May lose their drive licenses, afraid that they won’t be able to drive
	Punished by law	→In custody/Mandatory drug treatment isolation/Ordered to community drug rehabilitation/Every month they have to report to the Public Security Bureau/Undergo urine test/Use ID cards will be checked by police/Inconvenient to travel
	The expectation to live a normal life	→Proactively seek the supervision of public security organs/Hope to live a normal life

## Data Availability

The data presented in this study are not publicly available due to privacy concerns.
